# Visual working memory for connected 3D objects: effects of stimulus complexity, dimensionality and connectivity

**DOI:** 10.1186/s41235-022-00367-9

**Published:** 2022-02-19

**Authors:** Chuanxiuyue He, Peri Gunalp, Hauke S. Meyerhoff, Zoe Rathbun, Mike Stieff, Steven L. Franconeri, Mary Hegarty

**Affiliations:** 1 Department of Psychological and Brain Sciences, University of California, Santa Barbara, Santa Barbara, CA 93106 USA; 2 Leibniz-Institut Für Wissenmedien, Tübingen, Germany; 3 University of Illinois, Chicago, Chicago USA; 4 Northwestern University, Evanston, USA

## Abstract

Visual working memory (VWM) is typically measured using arrays of two-dimensional isolated stimuli with simple visual identities (e.g., color or shape), and these studies typically find strong capacity limits. Science, technology, engineering and mathematics (STEM) experts are tasked with reasoning with representations of three-dimensional (3D) connected objects, raising questions about whether those stimuli would be subject to the same limits. Here, we use a color change detection task to examine working memory capacity for 3D objects made up of differently colored cubes. Experiment 1a shows that increasing the number of parts of an object leads to less sensitivity to color changes, while change-irrelevant structural dimensionality (the number of dimensions into which parts of the structure extend) does not. Experiment 1b shows that sensitivity to color changes decreases similarly with increased complexity for multipart 3D connected objects and disconnected 2D squares, while sensitivity is slightly higher with 3D objects. Experiments 2a and 2b find that when other stimulus characteristics, such as size and visual angle, are controlled, change-irrelevant dimensionality and connectivity have no effect on performance. These results suggest that detecting color changes on 3D connected objects and on displays of isolated 2D stimuli are subject to similar set size effects and are not affected by dimensionality and connectivity when these properties are change-irrelevant, ruling out one possible explanation for scientists’ advantages in storing and manipulating representations of complex 3D objects.

**Supplementary Information:**

The online version contains supplementary material available at 10.1186/s41235-022-00367-9.

## Introduction

**Fig. 1:**
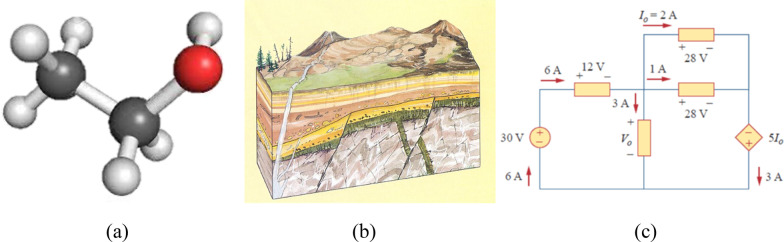
Examples of disciplinary representations, including **a** ball-and-stick (chemistry), **b** block (geology) and **c** circuit (engineering) diagrams

Constructing and maintaining representations of three-dimensional structures is important for success in science, technology, engineering and mathematics (STEM) disciplines (National Research Council, 2006). Disciplines such as chemistry, geology and engineering often require an ability to both understand the spatial properties of multipart objects and maintain representations of those objects (see Fig. 1). For example, in organic chemistry, two molecules with the same structure can have critically different properties depending on which atoms are bound to the structure. Scientists and their students can quickly detect changes in complex representations made up of many parts (Morphew et al., 2015). Research on visual working memory (VWM) suggests a capacity limit for simple items (such as shapes and colors) of around 3–4 (Brady, et al., 2011; Cowan, 2001; Luck & Vogel, 1997, 2013). Encoding and making judgments about STEM representations therefore seem to exceed working memory limits, raising questions about relative working memory demands of these types of representations.

**Fig. 2:**
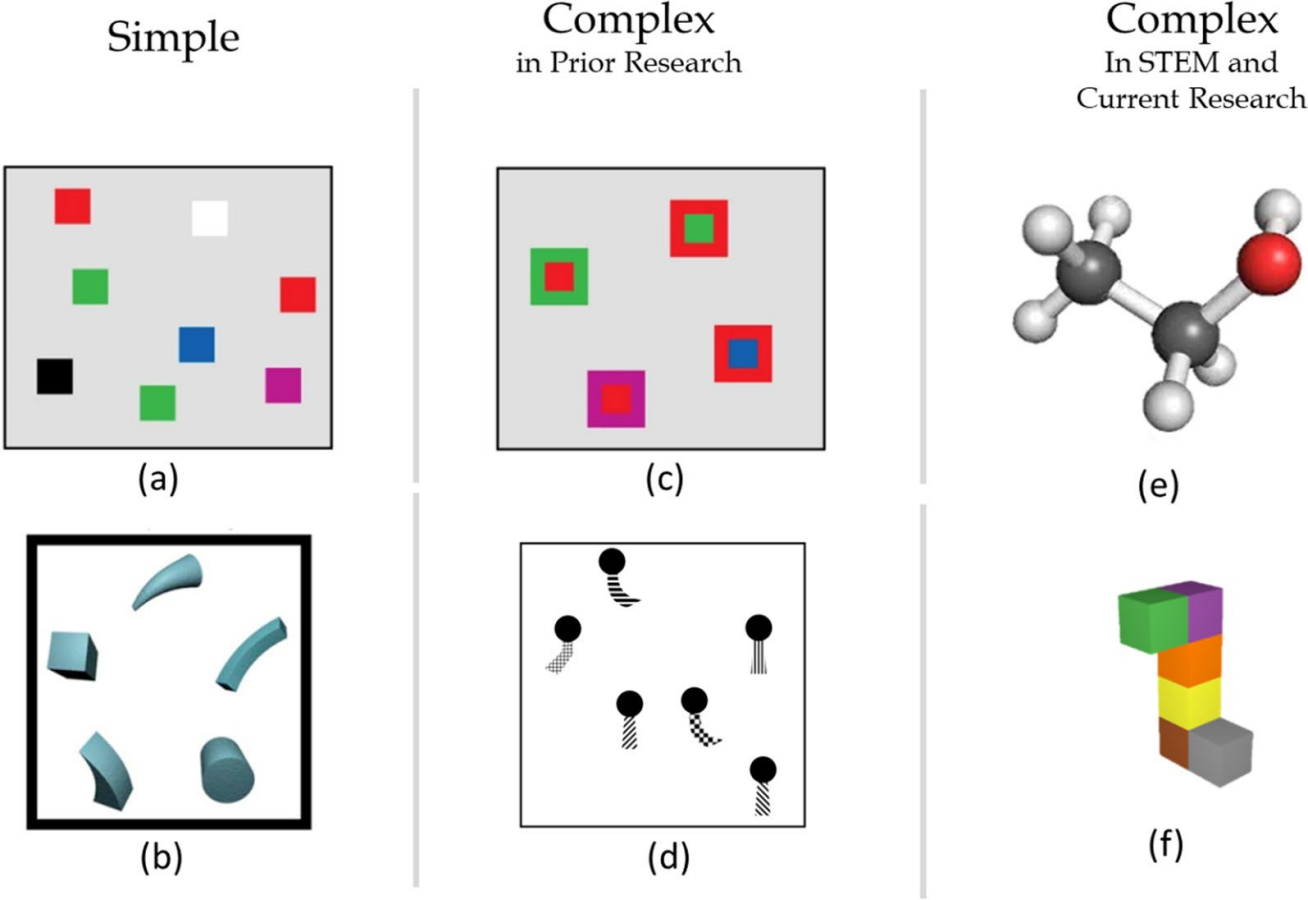
Stimuli comparisons. Examples of typical stimuli composed of (**a**) isolated 2D items (Luck & Vogel, 1997), (**b**) geons (Wood, 2009), (**c**) stimuli used for studying conjunction (Luck & Vogel, 1997), (**d**) two-part objects (Xu, 2006), (**e**) STEM  stimuli depicting molecules and (**f**) the stimuli in the current study. Three-dimensional (3D) multipart objects are different from 2D isolated items in terms of both dimensionality and connectivity

Working memory capacity is often measured by a change detection paradigm in which participants are shown a set of two stimuli separated by a brief delay and have to indicate whether the two displays are the same or different. As the number of items in the display increases beyond four, sensitivity to a change decreases (Brady et al., 2011; Luck & Vogel, 1997; Vogel et al., 2001). Visual working memory studies typically use displays composed of abstract, two-dimensional, isolated items (see Fig. 2a). In contrast, STEM representations, such as molecular representations (see Figs. 1a, 2e), often comprise complex three-dimensional objects made up of many connected parts. Here, we explore whether the set size effect found with displays of isolated objects also applies to representations of multipart objects and whether connectivity and dimensionality contribute to the apparent visual memory advantages for STEM representations. To preview our results, we find a set size effect for the number of parts of an object but no evidence that connectivity and dimensionality enhance visual working memory for STEM-like representations.

**Fig. 3:**
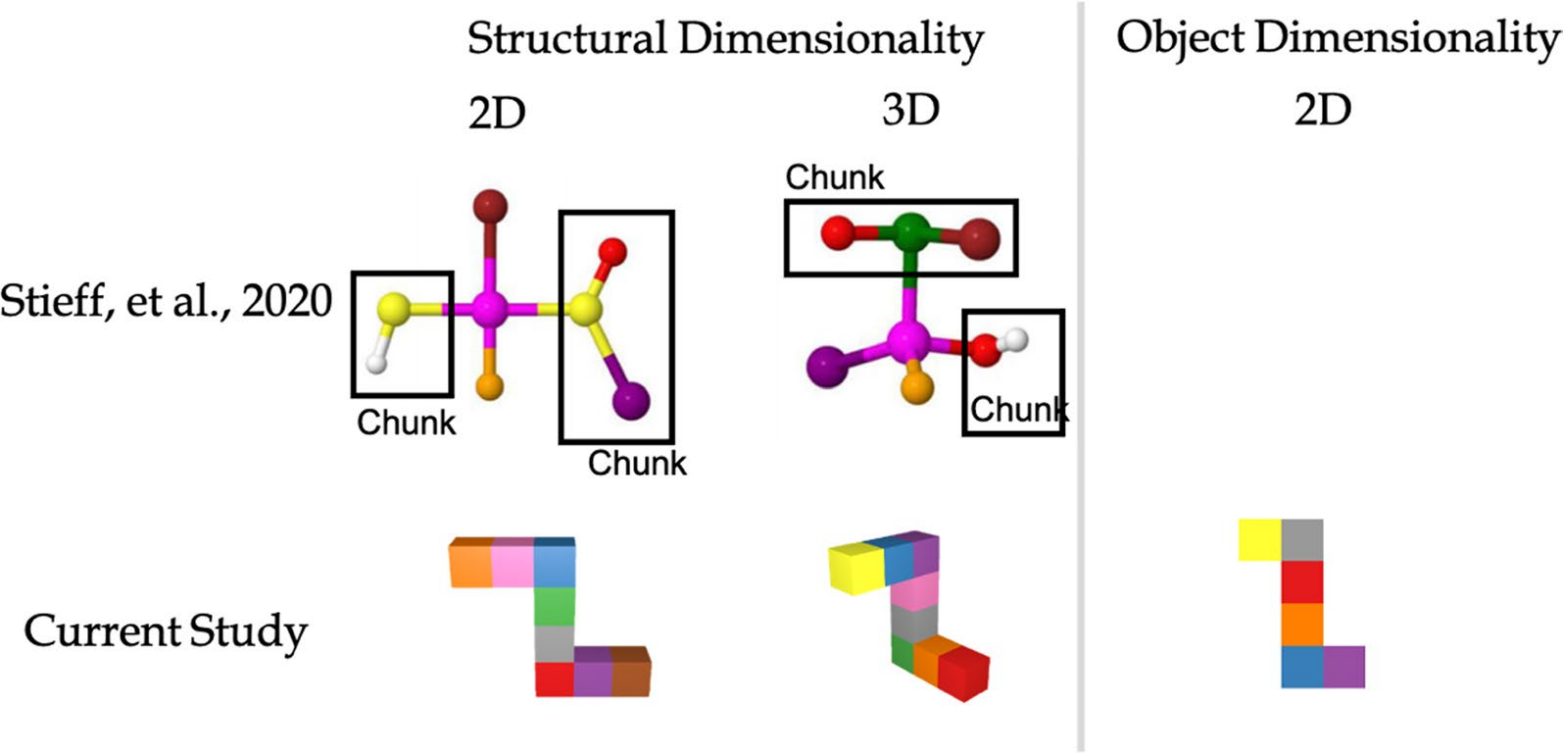
Comparisons between stimuli used in Stieff et al. (2020) and the current studies

Detecting the replacement of an atom in a chemical reaction is somewhat analogous to detecting the replacement of a color in a visual display in that different atoms are represented by different color changes in ball-and-stick molecular representations (see examples in Fig. 3). Inspired by this similarity, a recent study used a change detection task to examine visual working memory for these molecular representations (Stieff et al., 2020). Sensitivity to a change was better when the changes involved groups that correspond to recurring patterns of atoms in organic molecules (e.g., a hydroxyl group consisting of one oxygen atom bonded with a hydrogen atom) that formed visual “chunks” (see Fig. 3), compared to when the changes were to other atoms in the molecule. Interestingly, these effects were found for both organic chemistry students and students naive to chemistry, suggesting that students were sensitive to spatial groupings in these visual stimuli, regardless of their knowledge of the meaning of these groupings. This study motivated our current studies on other properties of the representations of 3D multipart objects that might affect working memory capacity.

### The present study

Here, we used a color change detection task to examine working memory for stimuli that have similar properties to molecular representations, in that they are complex 3D objects made up of connected solids, with different colors, and extending in different spatial dimensions. First, we examined how the *number of colored parts* of a single object affects performance in color change detection tasks when the number of parts exceeds two. All stimuli in the study by Stieff et al (2020) were made up of the same number of atoms, so that study could not establish how the number of parts of a single complex object affects visual working memory. The previous research on visual working memory has been conducted with 2-part stimuli of different colors (e.g., Luck & Vogel, 1997) or different color–shape combinations (e.g., Xu, 2006) (see Fig. 2c, d). However, these stimuli differ from the type of complex visual representations used in STEM in that they are 2D and contain isolated objects made up of only 2 parts. To our knowledge, basic research in visual cognition has not systematically examined whether the number of parts of a *single* object similarly affects performance in change detection tasks.

In addition to set size, we study the effects of *dimensionality* and *connectivity* on color change detection in complex objects. Molecular representations exhibit two different aspects of dimensionality: structural dimensionality and object dimensionality. Structural dimensionality refers to the number of dimensions into which a structure extends (one, two or three dimensions; the *x*-, *y*- and *z*-planes, see Fig. 3). Object dimensionality refers to the more traditional meaning of dimensionality; that is, the number of dimensions each stimulus unit has (e.g., 2D shapes vs. 3D geons). While dimensionality is irrelevant to a color change, a history of visual cognition has shown that 3D object-like stimuli are easier to perceive (Purcell & Stewart, 1991) and also enhance perception (Lanze, et al., 1982, 1985; Weisstein & Harris, 1974) and memory (Ankrum & Palmer, 1991) for line-drawn stimuli. Moreover, color change detection can be enhanced by including depth information in stereoscopic displays, when isolated colored squares are shown in different depth planes (Chunharas et al., 2019; Sarno et al., 2019; Xu & Nakayama, 2007). In contrast, Stieff et al. (2020) found that structural dimensionality (2D vs 3D) had no effect on change detection. However, because the changed elements of those ecologically valid ball-and-stick stimuli include multiple features such as the relative sizes of parts and angles between bonds in addition to color, detection of a single feature change could not be experimentally manipulated in that study without sacrificing ecological validity (see Fig. 3).

For connectivity, the previous research also suggests that accuracy in change detection tasks increases when features to be remembered are present on the same part of a multipart object (Xu, 2002b, 2006), are presented in close proximity to one another (e.g., Peterson & Berryhill, 2013; Wang et al., 2016) or are connected (Delvenne & Bruyer, 2006; Woodman et al., 2003; Xu, 2006). These effects are typically stronger for connectivity than for proximity (Woodman et al., 2003), possibly because the to-be-remembered information forms objects (Luck & Vogel, 1997) or facilitates the integration of feature conjunctions (Xu, 2006).

Overall, the present work examined questions important to STEM representations that no research to date has systematically investigated. First, we studied how the number of parts and dimensionality of a *single object* affect demands on working memory capacity (Experiment 1a & 1b) as measured by a color change detection task. Second, we examined the effects of two aspects of *dimensionality*: structural dimensionality (Experiment 1a) and object dimensionality (Experiments 1b), that is, stimulus properties that are irrelevant to a color change, on this task. Third, we examined the separate effects of change-irrelevant connectivity and dimensionality on color change detection (Experiments 2a and 2b).

## Experiment 1a

**Fig. 4:**
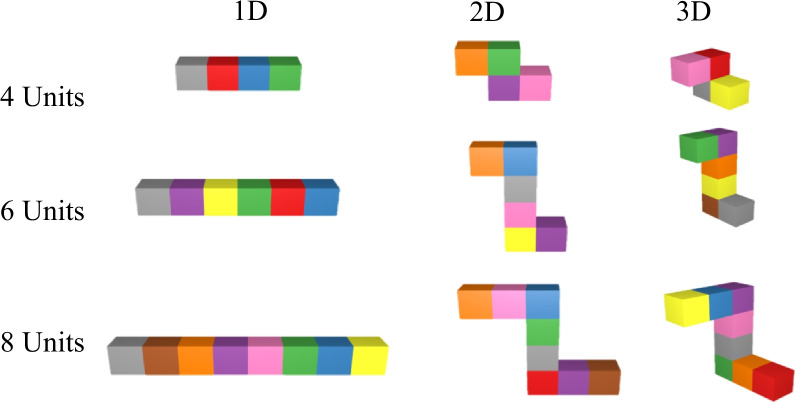
Examples of stimuli varying in structural dimensionality (extending in 1D, 2D or 3D) and complexity (made up of 4, 6 or 8 units)

In Experiment 1a, we varied two aspects of the stimuli: complexity (number of cube constituents) and structural dimensionality (the number of dimensions in which the cubes extended); see Fig. 4 for examples of stimuli. We predicted that set size effects found with isolated stimuli (Brady et al., 2011; Cowan, 2001; Luck & Vogel, 1997, 2013) would generalize to multipart objects, such that sensitivity to changes would decrease as the number of parts of an object increase. Structural dimensionality, however, could positively affect performance because participants can compress common features in a complex object by noting the locations of similar features (e.g., the red–orange–yellow group of colors were here, here and here), reducing the need to represent the actual color three times. This might boost performance even when the locations of those features are not explicitly relevant to the task (Brady & Alvarez, 2015a, 2015b; Brady et al., 2009a). It is less likely that this would happen in one-dimensional (1D) arrangements than in 2D and 3D arrangements, which provide progressively richer location representations and more items in close proximity to each other. According to this hypothesis (the *configural hypothesis*), sensitivity to a change should be lower in 1D objects than 2D or 3D objects.

### Method

#### Participants

Fifty-five students (35 female) participated. For all experiments, the participants were students from the University of California who had normal or corrected to normal vision and received course credit for participation. Participants were excluded from analysis if they had lower than 80% accuracy on a verbal concurrent task or if they had lower than chance (50%) accuracy on the change detection task. In Experiment 1a, four (female) students were excluded, for failure to reach the 80% criterion on the verbal concurrent task and one was excluded for lower than chance accuracy. An a priori power analysis for ANOVA using G*Power (Faul et al., 2007) with an alpha level of 0.05, power of 0.8 and an effect size of *f* = 0.176 (corresponding to a small effect size; *η*_*p*_^*2*^ = 0.03), indicated that our sample size (51) exceeded the minimum number of participants needed for this experiment to be sufficiently powered.

#### Materials

##### Apparatus

Stimuli were presented on a 24-inch ASUS VG248 monitor with an AMD Radeon T R7450 graphics card, 1920 × 1080 resolution, 60 Hz refresh rate and 8-bit depth.

##### Experimental task

A change detection task, programmed with the PsychoPy libraries (Pierce, 2007), was employed in which participants were shown a set of two stimuli separated by a brief delay and were asked to assess whether the second (test) stimulus differed from the first stimulus. To avoid use of verbal encoding strategies and verbal working memory (WM), a concurrent verbal task was employed. Stimuli were presented within a 20.6° region in the center of the computer monitor with a white background and viewed at a distance of approximately 70 cm.

##### Stimuli

The stimuli were pictures of objects consisting of connected colored cubes. Each cube within the stimulus had a unique color, which was selected randomly (without replacement) from a set of nine: red, orange, yellow, green, blue, purple, pink, brown and gray. Colors were selected using Color Brewer 2^1^ (*n* = 9, qualitative; Brewer, 2006) to ensure contrast between each of the values (RGB values are presented in Appendix). Objects were created and rendered using Blender version 2.78. Because each object was three-dimensional, the addition of depth and shading meant that there was variation in the luminance values of the colors of the objects.

Objects were composed of 4, 6 or 8 units. As the relative size of the cubes was preserved between conditions, objects with more units had a greater visual angle (4-unit objects had a maximum visual angle of 10.2°, for 6-unit objects, this visual angle was 15.9° and for 8-unit objects the maximum visual angle was 20.6°). The objects also varied in structural dimensionality. One-dimensional (1D) objects had cubes extending in only the *x*-coordinate plane, two-dimensional (2D) objects extended into both the *x* and *y* planes and three-dimensional (3D) objects extended in the *x*, *y* and *z* planes (see Fig. 4).

On half of the trials the sample and test stimuli were identical (except for a rotation of 10° clockwise or counterclockwise from the sample stimulus, to minimize the ability to detect changes by monitoring for local pixel changes). On the other half, they were identical except for a change in color of one single substituent cube and the same rotation. This change in color was selected randomly from the remaining colors in the nine-color set (i.e., a color not used in the sample stimulus). There were eight trials for each condition of the 3 (number of units) by 3 (structural dimensionality) by 2 (change, no change) factorial design for a total of 144 trials.

*Spatial ability measures* We also included two measures of spatial ability. Details of these measures and their correlations with performance are presented in Additional file 1.

*Color blindness measure* The Ishihara compatible pseudoisochromatic plate (PIPIC) color vision test (Waggoner, 2005) was used to test for color blindness.

#### Procedure

Participants were first administered the color blindness measure and then given instructions for the experimental task. They were first instructed on the verbal concurrent task and were told that they would be repeating four letters aloud throughout each trial. They were then given instructions on the experimental task, which explained that two structures would appear sequentially on the screen and, after seeing the second structure, their task was to indicate whether the two structures were the same or different. Participants were reminded that they should repeat the four letters throughout the trial and that they would be prompted to report the letters on randomly selected trials. Participants completed four practice trials, and if they were not confident in their understanding or performed poorly on these practice trials, they were asked to repeat them before proceeding.

**Fig. 5:**
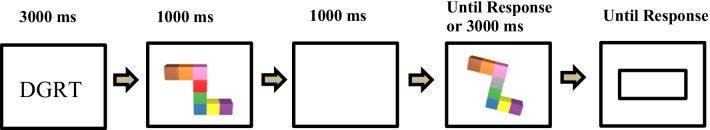
Procedure for the task in Experiment 1a. Participants first view a string of consonants, then a stimulus presented briefly, followed by a blank screen, and a second stimulus that may be either the same as or different from the first. After seeing the second stimulus, participants are to indicate whether the first and second stimuli are the same or different. On some trials, participants are also prompted to type in the consonant string presented at the beginning of the trial

The experimental procedure is shown in Fig. 5. In each trial, four randomly selected distinct consonants were first presented to the participants for 3000 ms. Participants were instructed to repeat this string of consonants aloud throughout the trial. After a 500-ms inter-stimulus interval, the sample stimulus was presented in the center of the screen for 1000 ms, followed by a 1000 ms retention interval. Finally, the test stimulus was presented (in the same location but rotated 10 degrees clockwise or counterclockwise from the sample stimulus, to minimize any memory contributions from similar retinotopic or afterimage-based representations) until the participant responded or until 3000 ms at which time the trial timed out. Participants responded by pressing one of two keys (“1” for different, “9” for same) on a standard keyboard for the visual working memory task and were given immediate feedback on their answer. On 20% of trials, they were prompted to report the string of consonants they had been repeating. On these trials, a box appeared in the center of the screen and participants typed the letters and again were given immediate feedback.

After completing the experimental task, participants were administered spatial ability measures (see Additional file 1) and an online questionnaire, which asked questions about strategies used to complete the structure comparison task and demographics.^2^

### Results and discussion

**Fig. 6:**
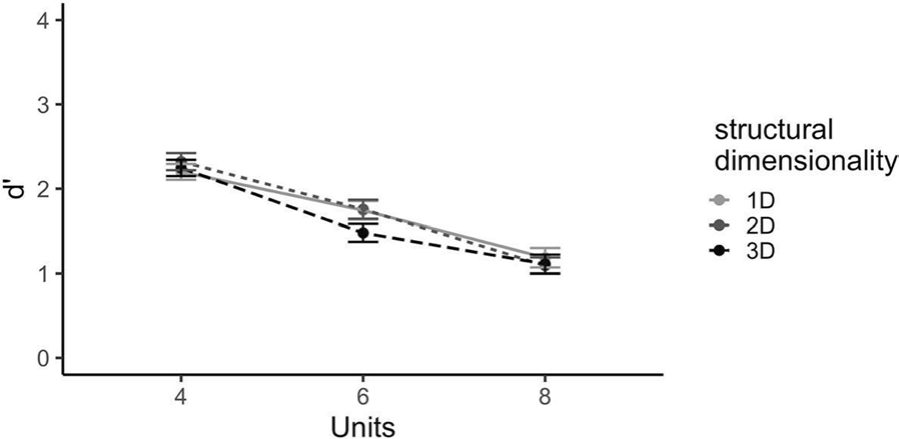
Performance for the Experimental Task in Experiment 1a. Sensitivity d' graphed as a function of number of parts and structural dimensionality (extension in 1, 2 or 3 dimensions). Standard error bars represent ± 1 SEM

Accuracy as a function of structural dimensionality, number of parts and target stimulus change is shown in Table 1. In all experiments in this study (see Tables 1, 2), participants had a positive response bias in all conditions, so additional analyses were conducted using d' (graphed in Figs. 6, 8, 10) as a measure of performance (see Additional file 1 for response times for all experiments).

**Table 1:** Means (standard errors in parentheses) for measures of accuracy Experiment 1a and 1b

	4 units		6 units		8 units	
	Change	No change	Change	No change	Change	No change
Exp. 1a: Structural dimensionality						
1D	.85 (.02)	.88 (.02)	.75 (.02)	.83 (.02)	.65 (.03)	.75 (.03)
2D	.86 (.02)	.90 (.02)	.70 (.03)	.88 (.02)	.56 (.03)	.81 (.02)
3D	.86 (.02)	.88 (.02)	.69 (.03)	.82 (.02)	.58 (.03)	.80 (.02)
Exp. 1b: Display type						
Square	.89 (.01)	.90 (.01)	.68 (.02)	.85 (.02)	.60 (.02)	.81 (.03)
Cube	.96 (.01)	.91 (.02)	.80 (.02)	.87 (.02)	.68 (.03)	.82 (.03)

**Table 2:** Means (standard errors in parentheses) for measures of accuracy for Experiments 2a and 2b

Display type	Connected		Disconnected	
	Change	No change	Change	No change
Experiment 2a				
2D	.76 (.03)	.84 (.03)	.77 (.03)	.83 (.03)
3D	.74 (.03)	.87 (.02)	.77 (.03)	.87 (.02)
Experiment 2b				
2D	.79 (.02)	.88 (.02)	.79 (.03)	.87 (.02)
3D	.76 (.03)	.89 (.02)	.78 (.02)	.85 (.02)

A 3 (number of parts: 4, 6, 8) $\times$ 3 (structural dimensionality: 1D, 2D, 3D) repeated-measures ANOVA on d’ found a large significant main effect of number of parts, *F*(2, 400) = 137.25, *p* < 0.001, *η*_*p*_^*2*^ = 0.41, no significant main effect of structural dimensionality, *p* = 0.20, and no significant interaction *p* = 0.23 (see Fig. 6). Notably, the Bayes Factor (BF_10_) for structural dimensionality was 0.059, indicating strong evidence that structural dimensionality had no effect, a result that is consistent with Stieff et al. (2020).

In sum, Experiment 1a showed that working memory capacity for our object-like stimuli was similar to working memory limits for simpler displays, in that sensitivity to a change decreased with more units in the structure. Moreover, we found that structural dimensionality, which is irrelevant to a color change, did not affect sensitivity. The Bayes Factor strongly supports the null hypothesis and there was no evidence for the alternative (*configural*) hypothesis.

## Experiment 1b

Experiment 1b directly compared the decline in sensitivity with more parts of a 3D multipart object to the decline in sensitivity with more isolated elements in a 2D display (typically used in visual working memory tasks). In this study, the same participants performed a change detection task with these two types of displays. We did not attempt to control other stimulus properties and the two types of stimuli varied in both structural dimensionality (the number of dimensions into which they extend) and object dimensionality (2D squares vs. 3D cubes).

### Method

#### Participants

Twenty-six undergraduate students (23 female) participated and one (female) was removed from subsequent analyses for less than 80% accuracy on the verbal secondary task. An a priori power analysis for ANOVA run using G*Power with an alpha level of 0.05, and power of 0.8, indicated that our sample size (*N* = 25) was sufficient to detect a small to medium effect size of *η*_*p*_^*2*^ = 0.05.

#### Materials

##### Experimental task

The same change detection paradigm (including the verbal working memory load task and display size) as in Experiment 1a was used.

##### Stimuli and design

**Fig. 7:**
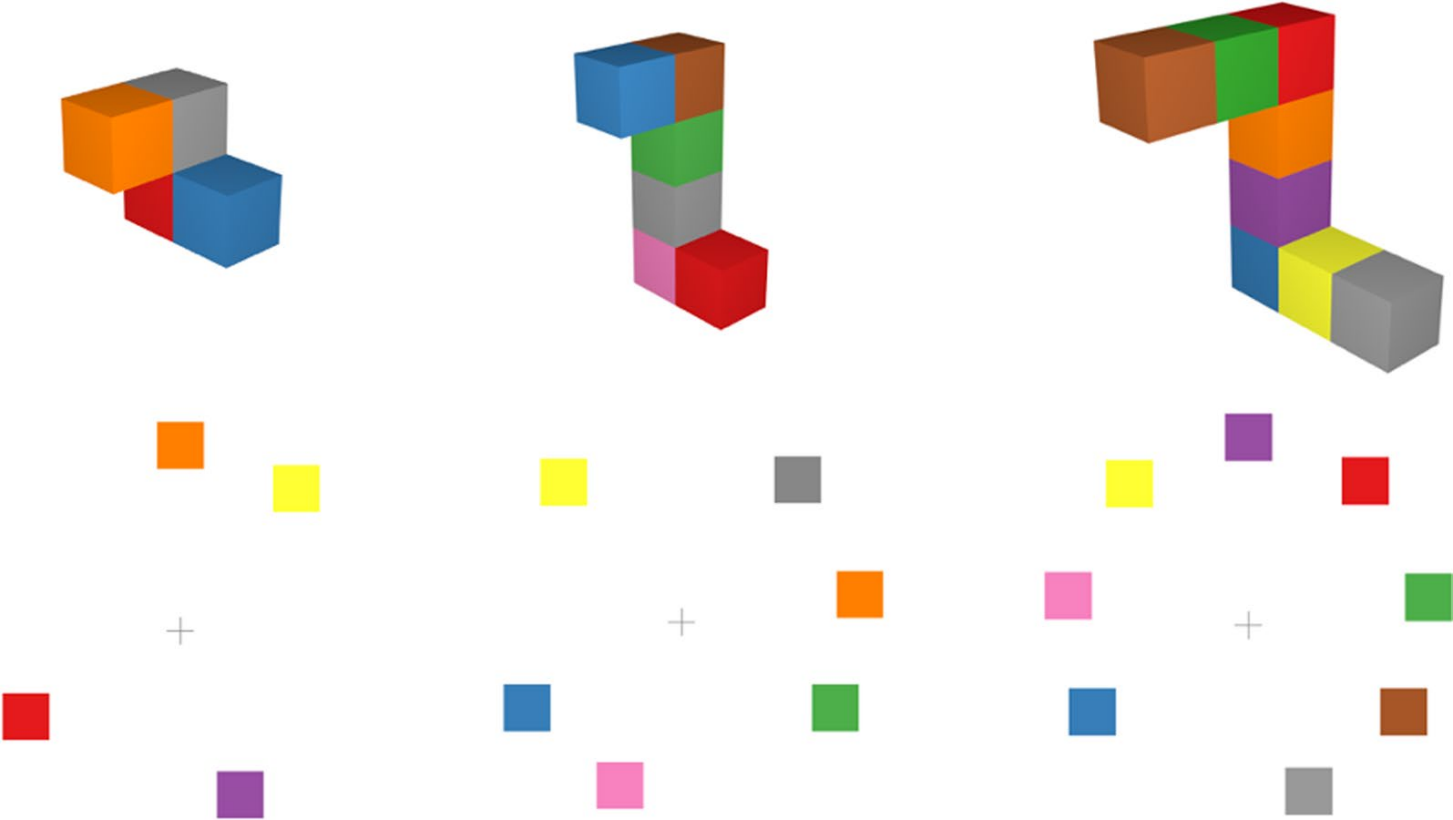
Sample displays of connected cubes (top) and disconnected squares (bottom) used in Experiment 1b

The experiment had a 2 (connected-cube, disconnected-square), by 3 (4, 6, 8 units), by 2 (change, no change) within-subjects design. All connected-cube stimuli extended in three dimensions (*x*, *y* and *z* planes), while disconnected-square stimuli extended in two dimensions. The disconnected-square stimuli were generated as follows: There were nine “spaces” in which a square could appear on the screen, and the locations of the squares were randomized between these nine spaces per trial (see Fig. 7). The spaces formed a circle with a visual angle of 12.5°. The colors of the squares were chosen from the same colors as used for the cube stimuli and pseudo-randomly assigned, such that each color appeared in each location roughly the same number of times. No colors were repeated within a single display, and when a cube/square changed from study to test stimulus, it changed to a different color not already in the display. There were 48 trials (24 change, 24 no change) for each level of stimulus (connected-cube, disconnected-square) by complexity (4, 6, 8 units) for a total of 288 trials.

#### Procedure

The procedure was similar to Experiment 1a (see Fig. 5) except that there was no 10-degree rotation due to the circular configuration of the 2D square stimuli. The experimental stimuli were presented in 12 blocks of 24 trials each, alternating between blocks of connected-cube and disconnected-square stimuli. Half of the participants began with a block of connected-cube stimuli and half began with a block of disconnected-square stimuli. Participants first completed the color blindness test and, after reading the instructions and successfully completing the practice trials for the first type of stimuli, proceeded to the first block. After the first block of test trials, participants completed practice trials for the other type of stimuli (connected-cube, disconnected-square) and then proceeded to the second block of trials. Participants completed the remaining 10 blocks of trials at their own pace.

### Results and discussion

**Fig. 8:**
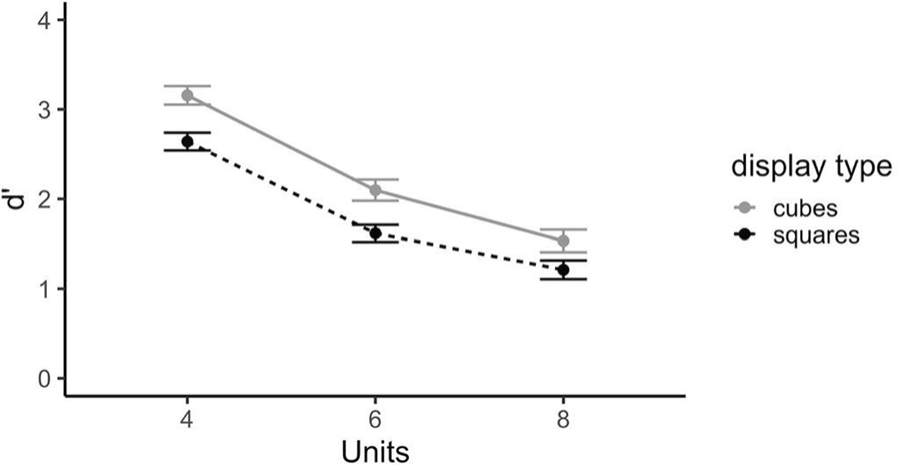
d′ as a function of display type (disconnected-squares versus objects made up of connected-cubes) and complexity (number of units in the display). Standard error bars represent ± 1 SEM

A 2 (display type: connected-cube, disconnected-square) by 3 (units: 4, 6, 8) repeated-measures ANOVA on d′ found a significant effect of display type, *F*(1, 120) = 34.96, *p* < 0.001, *η*_*p*_^*2*^ = 0.23 such that participants were significantly better at detecting changes in connected-cube displays (*M* = 2.26, SD = 0.89) than in disconnected-square displays (*M* = 1.82, SD = 0.78). There was also a significant effect of complexity, *F*(2, 120) = 146.91, *p* < 0.001, *η*_*p*_^*2*^ = 0.71, such that participants were significantly more sensitive to a change in four unit stimuli (*M* = 2.90, SD = 0.56) than either six-unit (*M* = 1.86, SD = 0.59) or eight-unit (*M* = 1.37, SD = 0.59) stimuli (see Fig. 8). Notably, there was no significant interaction, F(2, 120) = 0.66, *p* = 0.52 between these factors indicating that the number of visual features has the same relation to sensitivity for the two types of stimuli.

Experiment 1b showed a similar decline in performance for both types of stimuli. In addition, participants were more sensitive to the color changes with the connected-cube stimuli than with the disconnected-square stimuli. However, it is noted that these stimuli varied in multiple properties (i.e., dimensionality, connectivity, unit size and visual angle).

## Experiment 2

Experiments 2a and 2b were conducted to examine effects of stimulus dimensionality and connectivity on color change detection while controlling for other stimulus properties. Based on the previous research showing an advantage for 3D stimuli (Ankrum & Palmer, 1991; Lanze, et al., 1982, 1985; Purcell & Stewart, 1991; Weisstein & Harris, 1974), we tested the hypothesis that change detection would be enhanced for 3D objects (*dimensionality* hypothesis).

For the effects of connectivity, Experiment 2a more subtly manipulates this factor by either fully connecting objects or adding white borders around the cubes or squares of the objects, which have been shown to be sufficient to disrupt visual grouping of adjacent colors in visual search tasks (Yu et al., 2019). Experiment 2b tests a more extreme manipulation, by comparing those same fully connected objects to arrays of separate components (cubes or squares). On the basis of the previous research showing an advantage for connectivity (Delvenne & Bruyer, 2006; Luck & Vogel, 1997; Peterson & Berryhill, 2013; Wang et al., 2016; Woodman et al., 2003; Xu, 2002b, 2006), we predicted that sensitivity to changes would increase when components are connected. We refer to this hypothesis as the *connectivity* hypothesis.

## Experiments 2a

### Method

#### Participants

Twenty-one students (12 female) participated. Two (female) participants were excluded from the analysis because they had lower than 80% accuracy on the verbal concurrent task, leaving 19. With 19 participants, we can detect an effect size of *η*_*p*_^*2*^ = 0.08, which is much smaller than the effect size for the difference between disconnected 2D and connected 3D structures (*η*_*p*_^*2*^ = 0.23) in Experiment 1b.

#### Materials

##### Experimental task

The same change detection task procedure (including the verbal working memory load task) was used in Experiment 2. Stimuli were presented within a 20.6° region in the center of the computer monitor with a white background and viewed at approximately 70 cm.

##### Stimuli and design

**Fig. 9:**
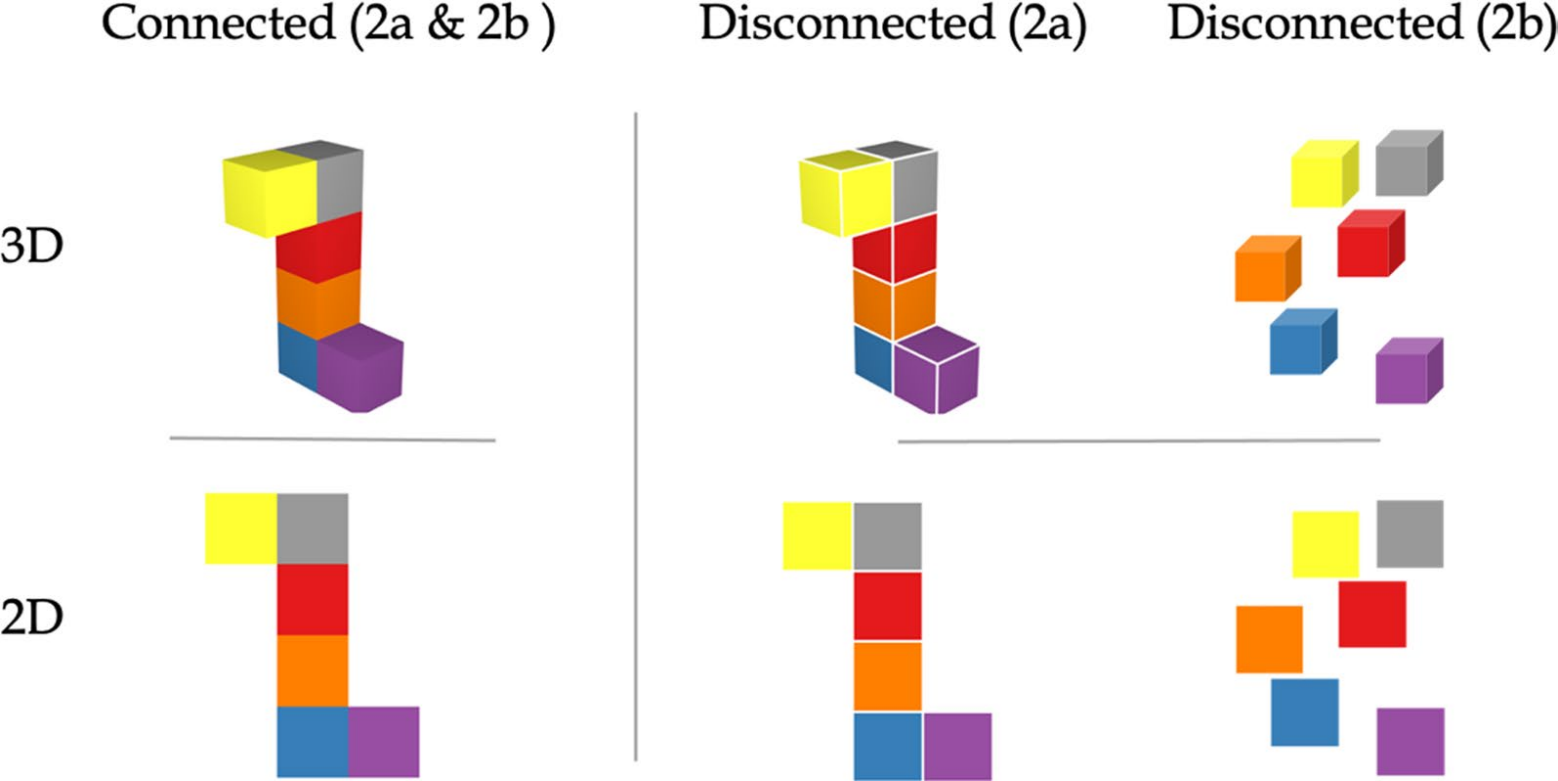
Sample stimuli used in Experiments 2a and 2b. The connected stimuli are the same in both experiments but disconnection was manipulated differently in the two experiments

The experiment had a 2 (connectivity: connected, disconnected), by 2 (dimensionality: 2D, 3D), by 2 (change, no change) within-subjects design. The connected 3D stimuli (see Fig. 9) were the same as the six-unit 3D stimuli used in Experiment 1b (see 6-unit cube stimuli in Fig. 7). The disconnected 3D stimuli were generated by adding a white border along the edge of each cube (see Fig. 9). The connected 2D stimuli had the same height as the connected 3D stimuli, so they were matched in vertical visual angle (but the width of 2D displays was 3.1 degree larger than the 3D display). The colors were assigned as in Experiments 1a and 1b and were matched across the four stimulus types. There were 48 trials (24 change, 24 no change) for each level of stimulus (2D, 3D) by connectivity (connected, disconnected) for a total of 192 trials.

#### Procedure

The procedure was the same as in Experiment 1b. Stimuli were presented in blocks of 24 trials, alternating between blocks of 2D and 3D stimuli, with stimuli randomly ordered within a block.

### Results and discussion

A 2 (dimensionality: 2D, 3D) by 2 (connectivity: connected, disconnected) repeated-measures ANOVA conducted on d’ data revealed no significant main effects or interactions of dimensionality and connectivity, all *p*’s > 0.36 (see Fig. 8). The Bayes Factor (BF_10_) for dimensionality is 0.331, indicating moderate evidence that dimensionality had no effect. The Bayes Factor (BF_10_) for connectivity is 0.298, indicating moderate evidence that connectivity had no effect. Thus there were no effects of connectivity or dimensionality when other stimulus factors were controlled.

## Experiment 2b

### Method

#### Participants

Twenty-five students (17 female) participated. Participants were drawn from the same subject pool as the previous experiments, but the experiment was conducted online due to the COVID-19 pandemic. Four participants (3 male) were excluded from the analysis because they had lower than 80% accuracy on the verbal concurrent task, leaving 21 participants in the final analysis. With 21 participants, we can detect an effect size of *η*_*p*_^*2*^ = 0.08 with an alpha level of 0.05, power of 0.8 (again much smaller than the effect observed in Experiment 1b).

#### Materials

##### Experimental task

The same change detection paradigm (including the concurrent verbal working memory load task) was employed. However, the experiment was administered via an online experiment hosting platform, Pavlovia (Grootswagers, 2020). The instructions were modified for online administration, although participants were given a phone number to call the experimenter if they had any questions. Also, four additional practice trials were added, for a total of eight practice trials. Finally, participants were prompted to retrieve the verbal memory load at the end of every trial (instead of only 20% of trials in previous experiments).

##### Stimuli

The experiment had a 2 (connectivity: connected, disconnected), by 2 (dimensionality: 2D, 3D), by 2 (change, no change) within-subjects design. The connected 3D and connected 2D stimuli were the same as in Experiments 1b and 2a. The disconnected 2D stimuli were generated such that the whole display had a bounding box defined by a rectangle with the same height and width of the bounding box of the connected 2D stimuli (i.e., controlling for visual angle). Six squares were randomly placed within the bounding box such that (1) they did not connect or intersect, (2) they were not aligned horizontally or vertically, and (3) the whole display was not smaller than the bounding box. These restrictions yielded four random spatial patterns of six squares or cubes, which were mirrored horizontally and vertically respectively to create an additional eight random patterns. The colors were assigned as in previous experiments and were matched across the four stimulus types. There were twenty-four trials for each condition of the 2 (dimensionality) by 2 (connectivity) by 2 (change or no change) design for a total of 192 trials.

#### Procedure

After giving informed consent, participants were given instructions for the first task, followed by eight practice trials and completed the first block of trials. Then they received instructions and practice trials for the second block of trials before completing these trials. The stimuli were presented in blocks of 24 trials, alternating between blocks of 3D displays and 2D displays. Half participants began with a block of 3D displays and half of them began with a block of 2D displays. Within each block, half of the stimuli were connected, and half of the stimuli were disconnected, with the order of trials randomized.

### Results and discussion

**Fig. 10:**
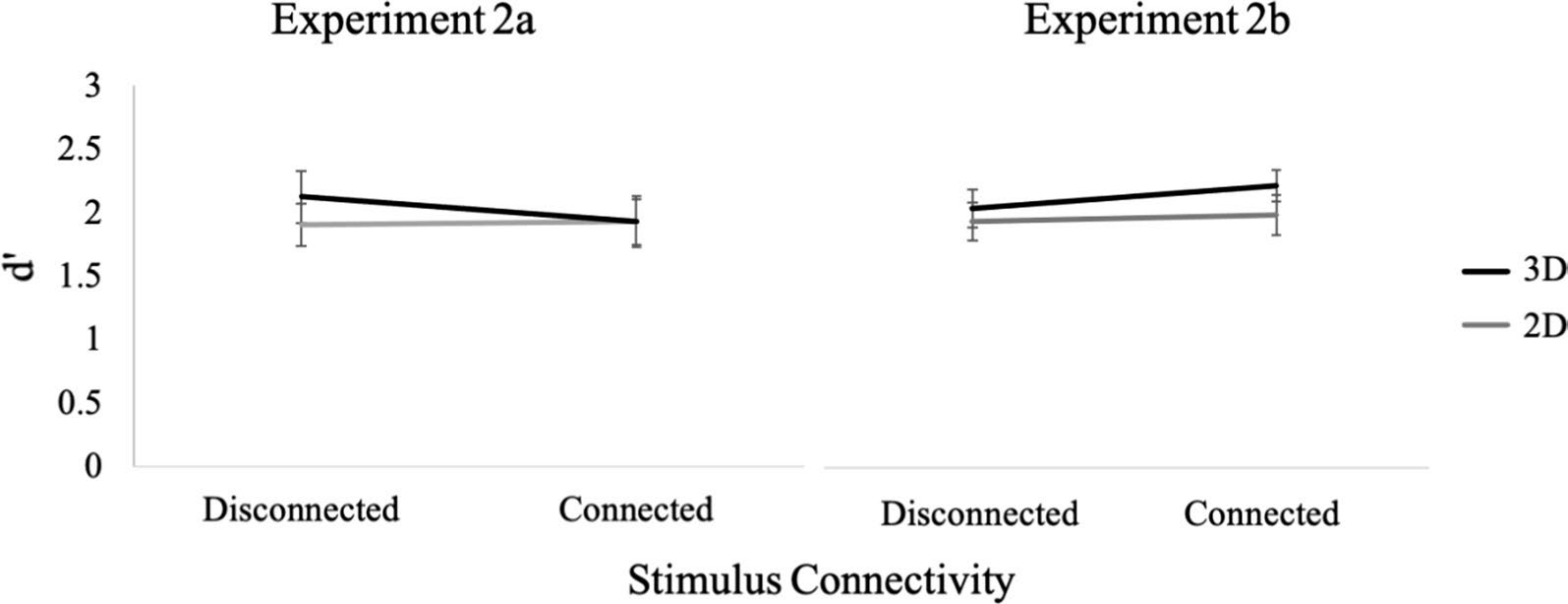
Sensitivity d′ as a function of stimulus connectivity and object dimensionality (2D, 3D). Results from Experiment 2a and 2b are shown on the left and right, respectively. Standard error bars represent ± 1 SEM

Accuracy as a function of dimensionality, connectivity and presence of a change is shown in Table 2. Participants had a positive response bias, so additional analyses were conducted using d' (graphed in Fig. 10) as a measure of performance.

A 2 (dimensionality: 2D, 3D) by 2 (connectivity: connected, disconnected) repeated-measures ANOVA conducted on d’ data revealed no significant main effects or interactions of dimensionality and connectivity, all *p*’s > 0.32. The Bayes Factor (BF_10_) for dimensionality is 0.347, indicating anecdotal evidence supporting the conclusion that change-irrelevant dimensionality had no effect on the color change detection. The Bayes Factor (BF_10_) for connectivity is 0.328, indicating moderate evidence that change -irrelevant connectivity had no effect either. Thus, there was no evidence in support of the connectivity hypothesis or the dimensionality hypothesis.

## General discussion

Across four experiments, we used a color change detection paradigm to examine visual working memory capacity for complex 3D objects made up of different numbers of visually distinct parts. In Experiments 1a and 1b we examined how color change detection is affected by the number of parts (varying from four to eight) of a single object; in Experiments 2a and 2b we examined how the color change detection for multipart objects compares to that for separate visual units (disconnected colored squares). Experiments 1a and 1b indicate that, when the task is color change detection, complex multipart objects are subject to similar working memory limits as displays made up of isolated 2D stimuli (Brady et al., 2011; Luck & Vogel, 1997, 2013). This conclusion is further supported by Experiments 2a and 2b, which indicate that being part of an object (that is, change-irrelevant dimensionality or connectivity), does not benefit color change detection.

Experiments 2a and 2b found no effects of connectivity on color change detection when other factors were controlled. This result contrasts with extant work finding that connectivity of objects improves memory capacity over and above proximity (Delvenne & Bruyer, 2006; Woodman et al., 2003; Xu, 2006). However, this previous research showed advantages of connectivity for integrating *different* feature dimensions (e.g., color and orientation). In contrast, our experiments examined the effects of connectivity on detection of features of the same type (color). Our results for these complex objects are thus consistent with the previous research on simpler objects that has shown memory advantages for connectivity only in displays where it can facilitate the integration of different types of features (e.g., color and shape) and not features of the same type (e.g., two colors) (Olson & Jiang, 2002; Parra, et al., 2011; Xu, 2002a).

Our results also provide new information about effects of stimulus dimensionality on visual working memory. Experiment 1a manipulated *structural dimensionality*: the number of dimensions into which the segments of the stimulus extended (1, 2 or 3) and found strong evidence for a null effect, a result that is congruent with those of Stieff et al. (2020) for STEM representations, indicating that differences in structural dimensionality do not effect color change detection independently of object complexity (number of units). Experiments 1b and 2 also manipulated *object dimensionality*: whether arrays were constructed with 2D squares or 3D cubes. Although Experiment 1b suggested a small effect of object dimensionality, this was no longer evident in Experiments 2a and 2b when other stimulus factors (size and visual angle) were controlled.

The lack of an effect for object dimensionality on color change detection contrasts with previous studies showing a benefit for working memory of colors in different depth planes (Chunharas et al., 2019; Sarno et al., 2019; Xu & Nakayama, 2007). In those studies, displays showing colored squares on planes in different depths may have enabled participants to use chunking or grouping strategies based on depth. In contrast, we presented one object whose entire structure extended in different dimensions. As acknowledged by Sarno et al. (2019), different types of representations involving varying depth cues may require different cognitive processes, and in fact, there can be an advantage of 2D in color change detection paradigms (Wood, 2011).

The 3D stimuli used in these experiments were designed to be analogous to representations in STEM disciplines such as those found in chemistry. Given our results, it seems unlikely that dimensionality and connectivity can account for the fact that chemistry experts can maintain and make judgments about complex visual representations made up of many visually distinct parts, when dimensionality and connectivity are irrelevant to the changes. There are several other possibilities that should be further explored. First, it is likely that chemists’ ability to make judgments about complex molecules is based on hard-won domain-specific knowledge that allows chunking of stimulus regularities in a visual display, as has been documented in other STEM disciplines, such as algebra (Goldstone et al., 2010) and physics (Morphew et al., 2015). Second, as noted, molecular representations may have other visual properties that enable visual chunking that even chemistry-naive students are sensitive to (Stieff et al, 2020). Third, while dimensionality and connectivity do not enhance detection of a color change, they might affect the detection of other changes, such as changes to the binding of color to structure, as in structural isomers which are composed of the same atoms but in a different configuration. Finally, the visual working memory paradigm may underestimate people’s capacities compared to more naturalistic tasks that scientists engage in, which do not require maintaining the complete representation of a complex stimulus in memory (Kristjansson & Draschkow, 2021).

These experiments help to fill a gap in the current VWM literature. To date, research has examined VWM for separated two-dimensional visual units (e.g., Brady & Alvarez, 2015b; Brady et al., 2011; Luck & Vogel, 1997), separated three-dimensional objects (Brady & Alvarez, 2015a; Wood, 2011) and real-world objects (Brady et al., 2009b; Kristjansson & Draschkow, 2021). Inspired by STEM representations, the present work extends this research to complex multicomponent stimuli and rules out a possible advantage for 3D object representations in detecting color changes, moving the field a step closer to understanding how VWM works for complex STEM representations.

### Supplementary Information

**Additional file 1:** Spatial ability measures in Experiment 1a and response time analyses for Experiment 1 & 2.

## Data Availability

Data and analysis script will be available on Github: https://github.com/CarolHeChuanxiuyue/Human_Working_Memory_Simple_Complex_Object_Analysis.git.

## Appendix

RGB values for colors used in stimuli (using Color Brewer 2).

Red: 228, 26, 28

Blue: 55, 126, 184

Green: 77, 175, 74

Purple: 152, 78, 163

Orange: 255, 127, 0

Yellow: 255, 255, 51

Brown: 166, 86, 40

Pink: 247, 129, 191

Gray: 153, 153, 153.

## Footnotes

1 Color Brewer is an online software program that produces color guidelines for cartography. This set consists of nine hues that are far in hue space, but also roughly perceptually equiluminant.

2 Strategy reports (e.g., using piecemeal vs. holistic strategies) were unrelated to performance on the task in any of our experiments. Moreover, some participants reported mixed strategies while about a third of strategy reports were uncodable, so strategies are not discussed further.
