# Supplementary material for: Visual working memory for connected 3D objects: effects of stimulus complexity, dimensionality and connectivity
Source: Cogn Res Princ Implic. 2022 Feb 19;7:19. doi: 10.1186/s41235-022-00367-9 (PMC8857738; doi:10.1186/s41235-022-00367-9)
Supplement: Supplementary file 1 — Additional file 1: Spatial ability measures in Experiment 1a and response time analyses for Experiment 1 & 2. [file 41235_2022_367_MOESM1_ESM.docx]

Supplementary Materials

**Spatial Ability Measures**

Given evidence that spatial abilities are important for success in STEM domains (e.g., Wai, Lubinski & Benbow, 2009), spatial ability measures were also measured in Experiment 1, in order to examine their relationship to performance on measures of visual working memory for complex objects. Measures of *visual* working memory have been found to be correlated with measures of *fluid* intelligence (Fukuda et al., 2010; Unsworth et al., 2014). Moreover *spatial* working memory is significantly correlated with performance on tests of *spatial* ability (Kane et al., 2004; Miyake et al., 2001; Shah & Miyake, 1996). However, we know of no research that has examined the correlation between *visual* working memory, as measured by change-detection tasks, and *spatial* ability.

The Paper Folding and Cube Comparisons tests were administered as measures of spatial ability. In the Paper Folding task (Ekstrom, Dermen, & Harman, 1976), participants are shown a depiction of a sheet of paper being folded, and a hole punched in one location. The task is to imagine what the configuration of holes would look like once the sheet of paper is unfolded and select which single depiction (of five answer choices) is correct. Participants complete two sets of 10 items and are allowed three minutes per set. The score is the number of items solved correctly minus one fourth of the number of items solved incorrectly. In the Cube Comparisons task (Ekstrom, et al., 1976), participants are shown depictions of two cubes with letters on each face of the cubes. No letters are repeated on a given cube. The participants are to determine if the two cubes are the same or different. Participants complete two sets of 21 items on each page (42 items total) and are allowed three minutes for each set. The score on the test is the number of items solved correctly minus the number solved incorrectly. Cronbach’s Alpha for the Paper Folding test was .84 and for the Cube Comparisons measure was .84, indicating good internal reliability for both.

A sensitivity power analysis indicated that given the sample size of n = 51, a one-tailed test, and power of .8 the study had the power to detect correlations of .33 or higher (correlations between visual working memory and fluid intelligence observed by Fukuda et. al, 2010 and Unsworth et. al, 2014 ranged from .33 to .44).

***Correlations with Spatial Ability Measures***

To examine the relation between visual working memory performance and spatial ability we calculated d’ across all trials in Experiment 1 and correlated this with measures of spatial ability. The Paper Folding (*M* = 11.18, *SD* = 4.59) and Cube Comparison (*M* = 16.48, *SD* = 9.14) scores were highly correlated, *r* (50) = .62, *p* < .001, and so were combined to a spatial ability composite score averaging their z-score. The correlation of spatial ability with d’, although positive, was not statistically significant, *r* (50) = .18, *p* = .18.

In sum, correlations between performance on our experimental task and measures of spatial ability were small and non-significant (despite power to detect a medium sized effect), suggesting that visual working memory capacity is not strongly related to spatial ability. These results provide no evidence that the correlation between spatial abilities and STEM performance (e.g., Wai et al., 2009; Uttal et al., 2013) can be accounted for by basic working memory capacity for complex objects made up of connected parts.

In interpreting this result, it is important to keep in mind that although the stimuli were visuo-spatial in nature, the changes to be detected in these stimuli were changes in color, so that the task measured capacity for visual, but not necessarily spatial features. The present results are consistent with research indicating a dissociation between visual and spatial working memory (e.g., Zimmer, 2008; Darling, et al., 2007; Hecker & Mapperson, 1997; Mecklinger & Muller, 1996). Ongoing research in our laboratories (He et al., 2020; Jardine et al, in preparation; Stieff et al. 2020) is examining the effects of color changes that are swaps rather than replacements of color, so that the change is to the spatial binding of color to spatial location. This ongoing research is also examining the effects of object rotations. With more spatially demanding tasks, we might observe a stronger correlation with spatial abilities.

**Response Times**

***Experiment 1a***

On 0.5% of trials, participants timed out. Analyses of response time (RT) data were conducted using only correctly answered trials completed before time-out (78.2% of trials). As shown in Table 1 there was no speed-accuracy tradeoff, in that participants took longer to respond to stimuli with more parts *F*(2, 857) = 38.41, *p* < .001, *η_p_²* = .08. There was a significant effect of structural dimensionality on response time *F*(2, 857) = 4.50, *p* = .01, *η_p_²* = .01, such that participants responded slowest to the 1D structures, providing limited support for the configural hypothesis.

*Table S1*

*Response Time (Standard Errors) in Second for Correctly Answered Trials in Experiment 1a*

|  |  |  | 4 units | | | 6 units | | | 8 units | | |  |
| --- | --- | --- | --- | --- | --- | --- | --- | --- | --- | --- | --- | --- |
|  |  | Structural Dimensionality | Change | No Change | | Change | No Change | | Change | No Change | |  |
| Response Time (sec) | | 1D | 0.98 (.03) | | 1.00 (.03) | 1.04 (.03) | | 1.06 (.03) | 1.06 (.03) | | 1.10 (.03) | |
|  |  | 2D | 0.95 (.03) | | 0.96 (.03) | 1.01 (.03) | | 1.01 (.03) | 1.06 (.04) | | 1.05 (.04) | |
|  |  | 3D | 0.96 (.03) | | 1.00 (.03) | 1.04 (.03) | | 1.06 (.03) | 1.07 (.04) | | 1.07 (.04) | |

***Experiment 1b***

As in Experiment 1a, in the present experiment participants timed out on 0.5% of trials. Time out trials and response times for incorrect trials were not analyzed here, leaving 81.2% trials. As indicated in Table S2, and consistent with Experiment 1a, there was no speed-accuracy trade-off for this experiment. A 3 (units: 4, 6, 8) by 2 (change type: change, no change) by 2 (display type: connected-cube, disconnected-square) repeated-measures ANOVA on response times indicated a significant main effect of units *F*(2, 269) = 33.95, *p* < .001, *η_p_²* = .20, such that participants were significantly faster when detecting changes in four unit displays (*M* = .98, *SE* = .02) than six unit (*M* = 1.04 *SE* = .02) or eight unit (*M* = 1.09, *SE* = .03) displays. There was also a significant effect of display type, *F*(1,269) = 8.00, *p* = .01, *η_p_²* = .03, such that participants were significantly faster when judging the cube displays (*M* = 1.02, *SE* = .02) than when judging the square displays (*M* = 1.05, *SE* = .02). Change type also had a significant effect, *F*(1,269) = 12.31, *p* = .001, *η_p_²* = .04, such that participants were significantly faster when judging the change trials (*M* = 1.02, *SE* = .02) than when judging the no-change trials (*M* = 1.06, *SD* = .02).

*Table S2*

*Response Time (Standard Errors) in Second for Correctly Answered Trials in Experiment 1b*

|  |  | | 4 units | | | | 6 units | | | | 8 units | | |  |
| --- | --- | --- | --- | --- | --- | --- | --- | --- | --- | --- | --- | --- | --- | --- |
|  | Display Type | | Change | No Change | | | Change | No Change | | | Change | No Change | |  |
| Disconnected-square | | 0.98 (.04) | | | 1.03 (.05) | 1.06 (.05) | | | 1.06 (.05) | 1.08 (.05) | | | 1.10 (.05) | |
| Connected-cube | | 0.92 (.04) | | | 0.98 (.05) | 0.99 (.04) | | | 1.06 (.05) | 1.07 (.05) | | | 1.10 (.05) | |

***Experiment 2a***

Timed out trials (0.5% of all trials) and response time for incorrect trials were not included in the analyses, leaving 80.7% remaining trials. A 2 (dimensionality) by 2 (connectivity) by 2 (change) repeated measures ANOVA conducted on response times indicated that responses to change trials were shorter (*M* = 0.97, *SE* = .02) than responses to no-change trials (*M* = 1.01, *SE* = .02), *F*(2, 129) = 11.79, *p* < .001, *η_p_²* = .08, but there were no effects of dimensionality or connectivity nor were there any significant interactions (all *p*’s > .17).

*Table S3*

*Response Time (Standard Errors) in Second for Correctly Answered Trials in Experiment 2a.*

|  | Connected | | Disconnected | |
| --- | --- | --- | --- | --- |
| Display Type | Change | No Change | Change | No Change |
| 2D | .96 (.03) | 1.00 (.04) | .96 (.03) | 1.02 (.04) |
| 3D | .98 (.03) | 1.03 (.04) | .99 (.03) | 1.00 (.03) |

***Experiment 2b***

Time out trials (1.6% of trials) and incorrect trials were not included in analyses response time analyses, leaving 82.7% remaining trials of the total trials. A 2 (dimensionality) by 2 (connectivity) by 2 (stimulus change) repeated measures ANOVA conducted on response times indicated that there were no significant main effects or interactions between factors *p*’s > .198 in all cases (descriptive statistics in Table S4).

*Table S3*

*Response Time (Standard Errors) in Second for Correctly Answered Trials in Experiment 2a.*

|  | Connected | | Disconnected | |
| --- | --- | --- | --- | --- |
| Display Type | Change | No Change | Change | No Change |
| 2D | .73 (.02) | .71 (.03) | .73 (.03) | .74 (.03) |
| 3D | .72 (.02) | .73 (.02) | .73 (.02) | .71 (.02) |

**Reference**

Darling, S., Della Sala, S., & Logie, R. H. (2007). Behavioural evidence for separating components within visuo-spatial working memory. *Cognitive Processing*, *8*(3), 175-181.

Ekstrom, R. B., Dermen, D., & Harman, H. H. (1976). *Manual for Kit of Factor-Referenced Cognitive Tests* (Vol. 102). Princeton, NJ: Educational Testing Service.

Fukuda, K., Vogel, E., Mayr, U., & Awh, E. (2010). Quantity, not quality: The relationship between fluid intelligence and working memory capacity. *Psychonomic Bulletin & Review*, *17*(5), 673-679.

He, C., Buonauro, D., Meyerhoff, H.S., Franconeri, S., Stieff, M., Hegarty, M. (2020, November). Compressing Symmetrical Structures Improves Change Detection. *Poster to be presented at the 61st Annual Meeting of the Psychonomics Society*.

Hecker, R., & Mapperson, B. (1997). Dissociation of visual and spatial processing in working memory. *Neuropsychologia*, *35*(5), 599-603.

Kane, M. J., Hambrick, D. Z., Tuholski, S. W., Wilhelm, O., Payne, T. W., & Engle, R. W. (2004). The generality of working memory capacity: a latent-variable approach to verbal and visuospatial memory span and reasoning. *Journal of Experimental Psychology: General*, *133*(2), 189-217.

Mecklinger, A., & Müller, N. (1996). Dissociations in the processing of “what” and “where” information in working memory: An event-related potential analysis. *Journal of Cognitive Neuroscience*, *8*(5), 453-473.

Miyake, A., Friedman, N. P., Rettinger, D. A., Shah, P., & Hegarty, M. (2001). How are visuospatial working memory, executive functioning, and spatial abilities related? A latent-variable analysis. *Journal of Experimental Psychology: General*, *130*(4), 621-640.

Shah, P., & Miyake, A. (1996). The separability of working memory resources for spatial thinking and language processing: An individual differences approach. *Journal of Experimental Psychology: General*, *125*(1), 4-27.

Unsworth, N., Fukuda, K., Awh, E., & Vogel, E. K. (2014). Working memory and fluid intelligence: Capacity, attention control, and secondary memory retrieval. *Cognitive Psychology*, *71*, 1-26.

Uttal, D. H., Meadow, N. G., Tipton, E., Hand, L. L., Alden, A. R., Warren, C., & Newcombe, N. S. (2013). The malleability of spatial skills: A meta-analysis of training studies. *Psychological Bulletin*, *139*(2), 352-402.

Wai, J., Lubinski, D., & Benbow, C. P. (2009). Spatial ability for STEM domains: Aligning over 50 years of cumulative psychological knowledge solidifies its importance. *Journal of Educational Psychology*, *101*(4), 817-835.

Zimmer, H. D. (2008). Visual and spatial working memory: from boxes to networks. *Neuroscience & Biobehavioral Reviews*, *32*(8), 1373-1395.
